# A Water Soluble CoQ_10_ Formulation Improves Intracellular Distribution and Promotes Mitochondrial Respiration in Cultured Cells

**DOI:** 10.1371/journal.pone.0033712

**Published:** 2012-03-14

**Authors:** Christian Bergamini, Noah Moruzzi, Antonella Sblendido, Giorgio Lenaz, Romana Fato

**Affiliations:** 1 Department of Biochemistry “G. Moruzzi”, University of Bologna, Bologna, Italy; 2 Scharper Therapeutics, Medical Department, Sesto S. Giovanni, Milano, Italy; University of Windsor, Canada

## Abstract

**Background:**

Mitochondria are both the cellular powerhouse and the major source of reactive oxygen species. Coenzyme Q_10_ plays a key role in mitochondrial energy production and is recognized as a powerful antioxidant. For these reasons it can be argued that higher mitochondrial ubiquinone levels may enhance the energy state and protect from oxidative stress. Despite the large number of clinical studies on the effect of CoQ_10_ supplementation, there are very few experimental data about the mitochondrial ubiquinone content and the cellular bioenergetic state after supplementation. Controversial clinical and in vitro results are mainly due to the high hydrophobicity of this compound, which reduces its bioavailability.

**Principal Findings:**

We measured the cellular and mitochondrial ubiquinone content in two cell lines (T67 and H9c2) after supplementation with a hydrophilic CoQ_10_ formulation (Qter®) and native CoQ_10_. Our results show that the water soluble formulation is more efficient in increasing ubiquinone levels. We have evaluated the bioenergetics effect of ubiquinone treatment, demonstrating that intracellular CoQ_10_ content after Qter supplementation positively correlates with an improved mitochondrial functionality (increased oxygen consumption rate, transmembrane potential, ATP synthesis) and resistance to oxidative stress.

**Conclusions:**

The improved cellular energy metabolism related to increased CoQ_10_ content represents a strong rationale for the clinical use of coenzyme Q_10_ and highlights the biological effects of Qter®, that make it the eligible CoQ_10_ formulation for the ubiquinone supplementation.

## Introduction

Coenzyme Q_10_ (CoQ_10_), also known as ubiquinone, is the predominant form of coenzyme Q in humans. It is a lipid-soluble molecule composed of a redox active quinone ring and a hydrophobic tail. In the mitochondrial respiratory chain it acts as a mobile electron transporter and is a cofactor of uncoupling proteins [1]. When reduced, it is a powerful antioxidant that prevents oxidative damage by free radicals, including oxidation of lipids within the mitochondrial membrane [2]. There is evidence that CoQ_10_ affects the expression of hundreds of human genes involved in cell signaling, metabolism and nutrient transport [3] and it may have anti-inflammatory effects via gene expression modification [4]. Heart, kidney, brain and liver tissues show the highest concentration of CoQ_10_, which is endogenously synthesized and in small part assimilated from the diet [5].

The fundamental role of ubiquinone in mitochondrial function and cellular bioenergetics should make it the main dietary supplement in situations where its production is inadequate [6] or in pathological conditions where alterations of mitochondrial enzymes involved in CoQ_10_ redox mechanisms occur [7] such as cardiovascular disease [8], metabolic diseases [9], oxidative stress and aging [10].

The rationale for CoQ_10_ therapy is supported by the evidence of decreasing CoQ_10_ levels with age in human and animal tissues, further suggesting a potential therapeutic role in age-related neurodegenerative disorders [11], [12], [13].

Despite these potential beneficial effects on disorders related to mitochondrial dysfunction, clinical studies showed controversial results. The use of CoQ_10_ in neurodegenerative disorders failed to demonstrate any positive result in patients with Huntington's [14] and Parkinson's diseases [15] or amyotrophic lateral sclerosis [16]. Controversial results were observed in primary hypertension and statin induced myalgia [17] as well.

Therapeutic applications of CoQ_10_ are greatly limited by its poor bio-availability, due to its lack of solubility in aqueous media. A recent study demonstrated that, in rats, only 3% of orally administered CoQ_10_ can be absorbed [18]. Several advancements have been made to enhance the bioavailability of CoQ_10_ using various approaches like size reduction, solubility enhancement (by solid dispersion, prodrug, complexation, ionization) and use of novel drug carriers such as liposomes, microspheres, nanoparticles, nanoemulsions and self-emulsifying systems [19], [20]. For an updated review see: Villalba et al. [21].

The goal of the present study was to increase the mitochondrial content of CoQ_10_ in cultured cells (T67 and H9c2 cell lines), in order to improve their bioenergetics parameters. For this purpose we supplemented cultured cells with a water-soluble CoQ_10_ formulation Qter®, obtained by terclatration of native CoQ_10_. Mitochondrial respiration rate supported by different substrates (glucose, glutamate/malate and succinate/glycerol 3-phosphate), cellular ATP and protein content were analyzed to describe the energy state of the cells. The antioxidant properties of CoQ_10_ and Qter® were assessed by means of fluorogenic probes (DCFDA and MitoSOX red). Moreover, we wanted to highlight the importance of a correct ubiquinone insertion into the mitochondrial membrane that depends mainly on its bioavailability, rather than on the administered amount.

## Results

### Titration of Coenzyme Q10 uptake

**Figure 1:**
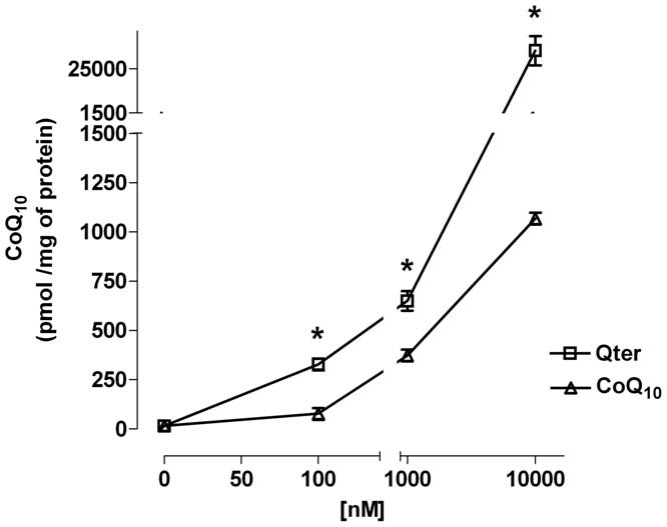
Titration of CoQ_10_ uptake in H9c2 cells. H9c2 cells were treated with different concentrations of native CoQ_10_ or Qter® dissolved in colture medium. After 24 hours cells were carefully washed with PBS and CoQ_10_ was extracted with exane/ethanol 5∶2 from whole cells and its concentration was determined by HPLC analysis. Data are normalized on total cellular protein content. Values are means ± S.D., n = 3, * p<0.001 vs. native CoQ_10_ treated samples.

**Figure 2:**
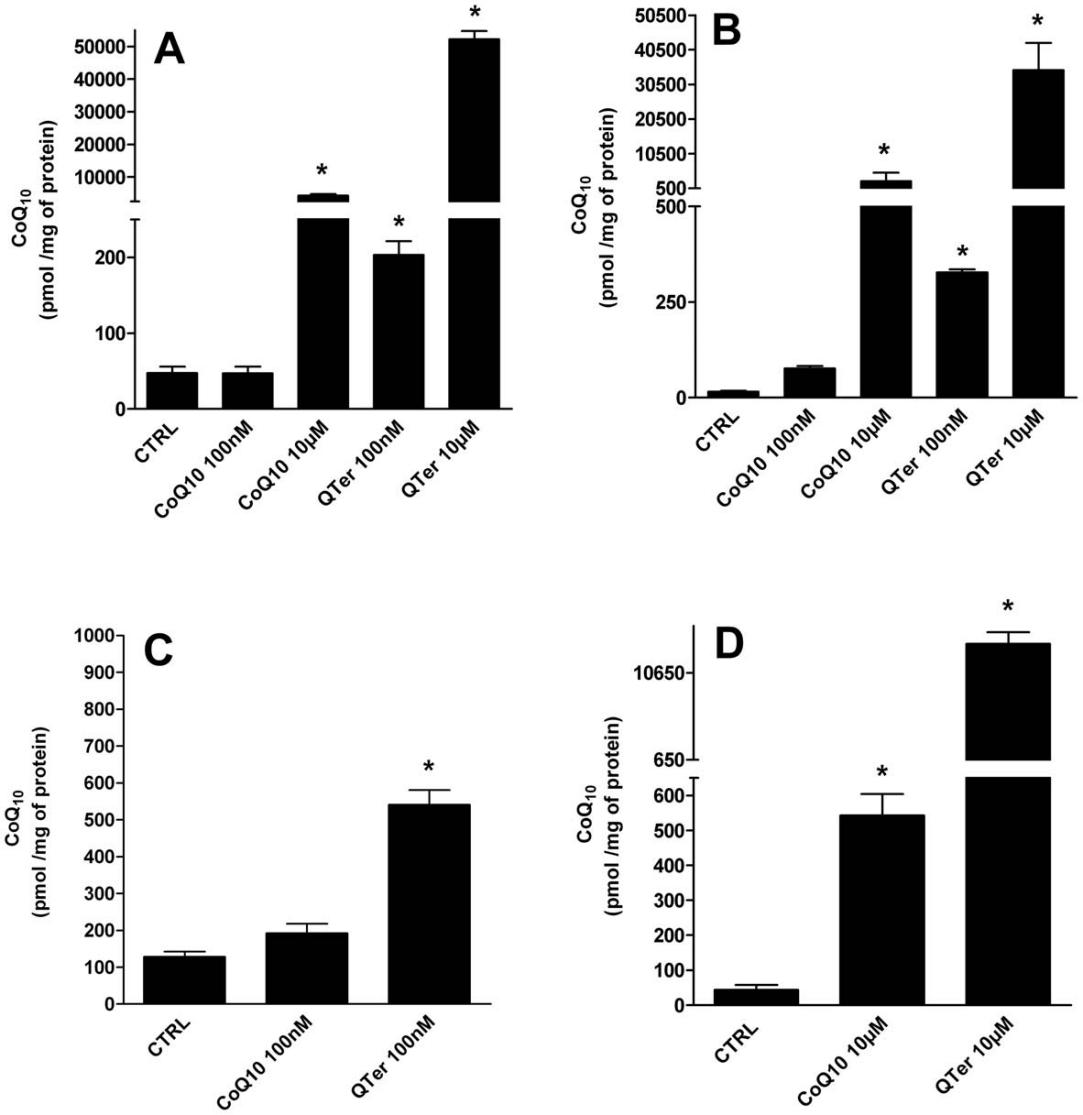
HPLC determination of CoQ10 cellular content. HPLC determination of CoQ_10_ cellular content in T67 cells (A) and H9c2 (B) treated for 24 hours with native CoQ_10_ or Qter® at different concentrations (100 nM and 10 µM). Coenzyme Q_10_ content was measured also in isolated mitochondria from T67 (C) and H9c2 (D) cells. Data were normalized on total cellular and mitochondrial protein content. Values are means ± S.D., n = 3, * p<0.001 vs. control.

Preliminary experiments were designed to establish the adequate concentration of ubiquinone, and to evaluate the effects of the pharmaceutically inactive matrix used to terclatrate CoQ_10_. Treatment for 24 hours with CoQ_10_ concentrations ranging from 10 nM to 10 µM, or vehicle, did not significantly alter the cell viability as confirmed by trypan blue exclusion method (data not shown). As shown in Figure 1 CoQ_10_ uptake was constantly more efficient in Qter® treated cells compared to native CoQ_10_. In particular 100 nM Qter® appeared to be sufficient to significantly increase CoQ_10_ content, while micro molar concentrations of native CoQ_10_ had to be administered to achieve a similar concentration. In addition, we found that mitochondrial CoQ_10_ content in cells treated with 100 nM Qter® was similar to that measured in cells treated with 10 µM native CoQ_10_ (Fig. 2C and 2D).

### Cellular and mitochondrial distribution of CoQ_10_

Figure 2A and 2B show the CoQ_10_ intracellular concentrations following treatment with different formulations and concentrations of ubiquinone. At the lowest concentration tested (100 nM) native CoQ_10_ was not able to significantly increase the cellular amount of CoQ_10_ in both cell lines, but only with a higher concentration (10 µM) it was possible to achieve significant results (p≤0.001). On the other hand the increase of cellular CoQ_10_ amount is at least 4 fold higher with Qter® compared to native CoQ_10_ and significant effects (p≤0.001) are already visible at the lower concentration (100 nM), that has no effect for native CoQ_10_. In human astrocytoma mitochondria 100 nM Qter® treatment showed a 3 fold increase of mitochondrial CoQ_10_ content compared to the same concentration of native CoQ_10_ (Figure 2C). A similar result was observed in isolated mitochondria from embryonic rat heart cells (Figure 2D) where mitochondrial CoQ_10_ content was 20-fold higher with 10 µM of Qter® compared to the same amount of native CoQ_10_ (p≤0.001).

### Mitochondrial respiration and cellular ATP content

We tested the effects of CoQ_10_ supplementation on cellular respiration (Table 1).

**Table 1:** Respiratory rates of intact H9c2 and T67 cells treated for 24 hours with 100 nM native CoQ_10_ or Qter®.

	EndogenousRespiration*nmoles O_2_ min^−1^/10^6^cells*	UncoupledRespiration*nmoles O_2_ min^−1^/10^6^cells*
*T67*		
Control	3.49±0.42	4.27±0.19
CoQ_10_	3.84±0.19	4.26±0.55
Qter	3.60±0.28	4.92±0.41*
*H9c2*		
Control	6.75±1.34	19.23±2.60
CoQ_10_	7.40±0.35	17.05±0.36
Qter	7.35±0.63	25.85±3.28*

Respirometric analyses were performed under endogenous and uncoupled conditions. The maximal uncoupled respiration was measured in the presence of 500 nM FCCP. Respiratory rates are expressed as nmoles O_2_ min^−1^/10^6^cells ± S.D. from at least three independent experiments. *p<0.05 vs. control.

Glucose supported oxygen consumption rates (OCR) were measured in intact cells in the presence and absence of 500 nM of the uncoupler FCCP. No effect on endogenous cell respiration was observed when both cell lines were treated with 100 nM native CoQ_10_ or Qter®, while the uncoupled OCR were significantly increased only by 100 nM Qter® in the two cell lines tested. A similar trend could be observed in permeabilized cells (Table 2). The state 3 respiration sustained by glutamate/malate was increased by treatment with Qter® and not with native CoQ_10_ in H9c2 cells (*p*<0.05 vs. control). Succinate/glycerol 3-phosphate supported oxygen consumption was significantly increased in both cell lines only after Qter® supplementation (*p*<0.001 vs. control). To test whether similar effects could be achieved raising the amount of CoQ_10_ administered, we treated the cells with higher amounts of ubiquinone (10 µM) but we couldn't observe any improvement in the endogenous cellular respiration rate even in presence of FCCP, as reported in Table 3. In these conditions native CoQ_10_ treatment decreased the rate of uncoupled respiration.

**Table 2:** Respiratory rates of permeabilized H9c2 and T67 cells treated for 24 hours with 100 nM native CoQ_10_ or Qter®.

	Glutamate/Malate*nmoles O_2_ min^−1^/10^6^cells*	Succinate/Glycerol 3-phosphate*nmoles O_2_ min^−1^/10^6^cells*
*T67*		
Control	3.92±1.02	5.26±1.31
CoQ_10_	3.26±1.05	6.25±0.92
QTer	4.3±1.14	7.85±0.07 *
*H9c2*		
Control	8.81±0.16	12.60±0.70
CoQ_10_	9.20±0.14	13.76±0.28
QTer	10.32±0.66*	16.95±0.21**

Respirometric analyses were performed in the presence of 5 mM Glutamate/Malate or 12,5 mM Succinate/Glycerol 3-phosphate. Respiratory rates are expressed as nmoles O_2_ min^−1^/10^6^ cells ± S.D. from at least three independent experiments, *p<0.05 vs. control. **p<0.001 vs. control.

**Table 3:** Percentage of respiratory rates measured in T67 cells treated for 24 hours with 10 µM native CoQ_10_ or Qter®.

	% of endogenous respiration rate	
Cell treatment	- FCCP	+500 nM FCCP
No treatment	100±7.2	131±13
10 µM CoQ_10_	100±8.6	84±8,6
10 µM Qter	100±1.1	102±14

Respirometric analyses were performed under endogenous and uncoupled conditions (500 nM FCCP). Respiratory rates in the presence of FCCP are expressed as percentage of oxygen consumption respect to the endogenous respiration ± S.D.

**Figure 3:**
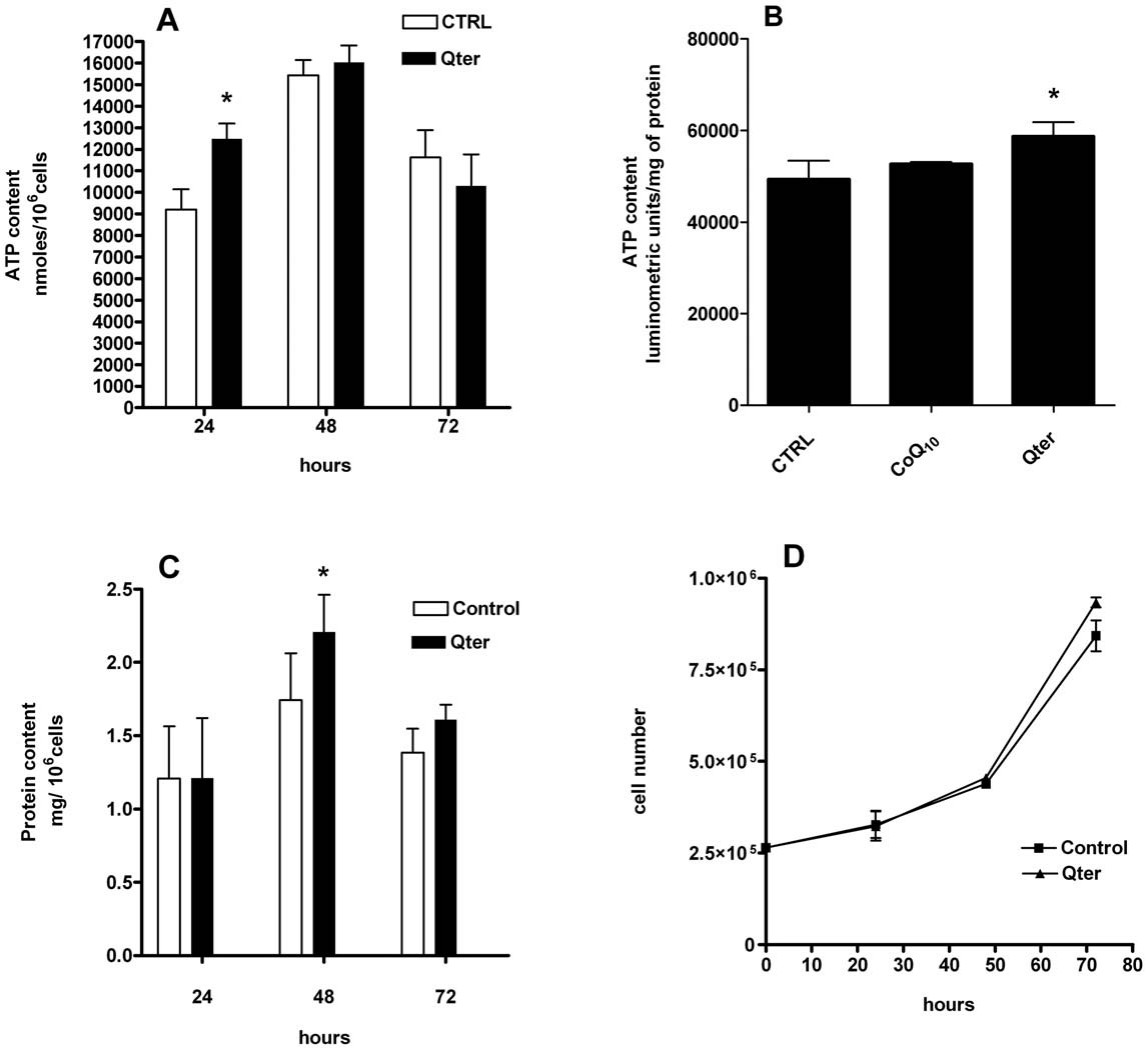
Effect of Qter® treatment on ATP, protein content and cell growth in H9c2 cells. H9c2 cells were treated up to 72 hours with 100 nM Qter® and the ATP content was measured at 24, 48 and 72 hours by HPLC analysis (A). Panel B shows the intracellular ATP content after 24 hours treatment with 100 nM Qter® or native CoQ_10_, measured using luminescence ATP detection assay. Data are reported as arbitrary luminometric units and normalized on total protein content. (Values are means ± S.D.,n = 5, * p≤0.01 vs control). H9c2 cells treated with 100 nM Qter up to 72 hours were assayed for protein content at 24, 48 and 72 hours. Protein content was evaluated by Lowry method (C), (Values are means ± S.D., n = 5, * p≤0.05 vs. control). Cell growth was assessed by trypan blue exclusion method (D).

Effects on ATP and protein content and cellular growth were analyzed at 24, 48 and 72 hours after 100 nM Qter® supplementation (Fig. 3). HPLC analysis showed that the ATP content was significantly higher in H9c2 cells treated with 100 nM Qter® for 24 h compared to the control, while no differences were observable at later time points (Figure 3A). ATP increase was confirmed also by luminometric assay in cells treated with the same amount of Qter® while native CoQ_10_ failed to show any effect (Figure 3B). Interestingly, cellular protein content was normal at 24 hours, and after 48 h cells treated with 100 nM Qter® showed higher protein content (Figure 3C). Qter® administration has no effect on cell growth (Figure 3D).

### Oxidative stress

It is well known that mitochondrial impairment is the principal source of ROS in the cell, moreover ROS production may be stimulated by treatment with radicals initiators such as tert-butyl hydroperoxide (TBH). To assess the ROS levels in biological samples we utilized two fluorescent probes: DCFDA and MitoSOX Red.

**Figure 4:**
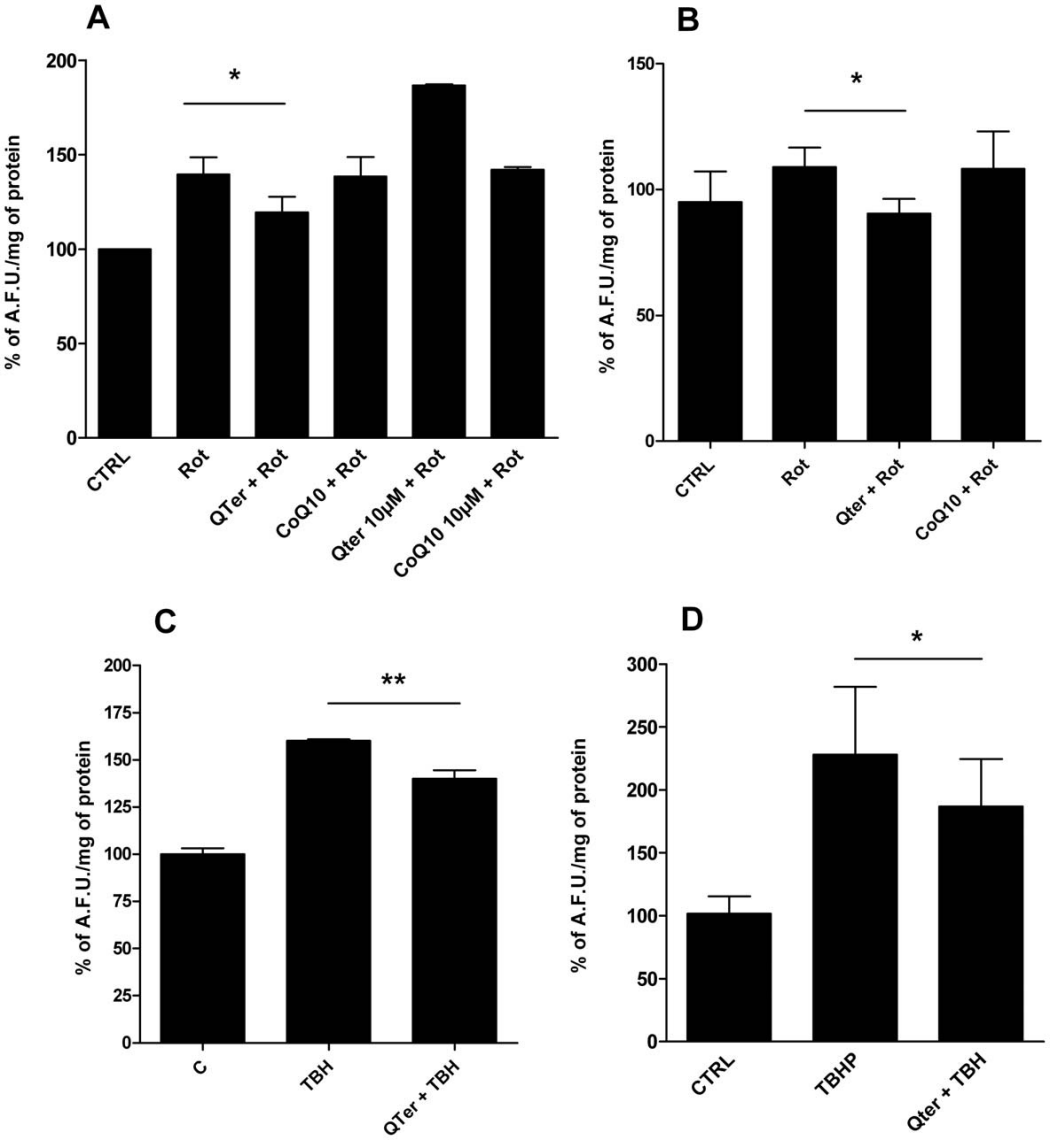
Effects of CoQ_10_ supplementation on oxidative stress induced by Rotenone and t-Butyl hydroperoxide (TBH). ROS were detected following DCFDA fluorescence in cells treated for 24 hours with 100 nM or 10 uM native CoQ_10_ or Qter®. ROS were induced by 48 hours treatment with 100 nM Rotenone in T67 (A) and H9c2 cells (B) or by 30 minutes exposure to 100 µM TBH in T67 cells (C) and H9c2 cells (D). Data are the mean ± S.D. of at least three different determinations and are expressed as arbitrary fluorescence units (A.F.U.) normalized on protein content. Protein content was evaluated by Lowry method. Asterisks refer to the statistically significant decrease of ROS production in Rotenone/TBH treated samples supplemented with quinones (n = 5, * p≤0.05); ** p≤0.001).

Figure 4 shows the protective effect of Qter® against oxidative stress induced by 100 nM Rotenone (a specific Complex I inhibitor) in T67 cells (Fig. 4A) and H9c2 (Fig. 4B) or 100 µM TBH in T67 cells (Fig. 4C) and H9c2 (Fig. 4D).

Pre-treating cells for 24 hours with 100 nM Qter® reduces the total amounts of cellular ROS (Fig. 4A, 4B, 4C, 4D), whereas in the same conditions native CoQ_10_ is less efficient. Moreover, cellular pre-treatment with higher Qter® concentration (10 µM), not only failed to improve protection against ROS production, but increased the oxidative stress. (Fig. 4A)

**Figure 5:**
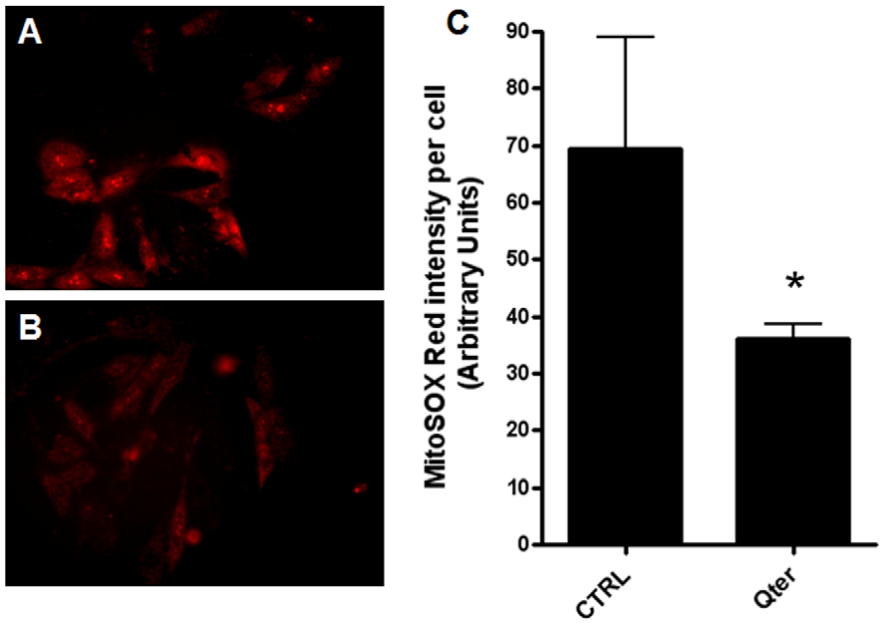
Analysis of physiological mitochondrial superoxide production using MitoSOX Red. The representative fluorescence images showed the oxidized MitoSOX fluorescence signal in control H9c2 cells (A) and H9c2 cells following 24 treatment with 100 nM Qter® (B). The fluorescence intensity reported in panel C was quantified by Image J software. Values are presented as means ± SD; *n* = 20. * p≤0.001.

**Figure 6:**
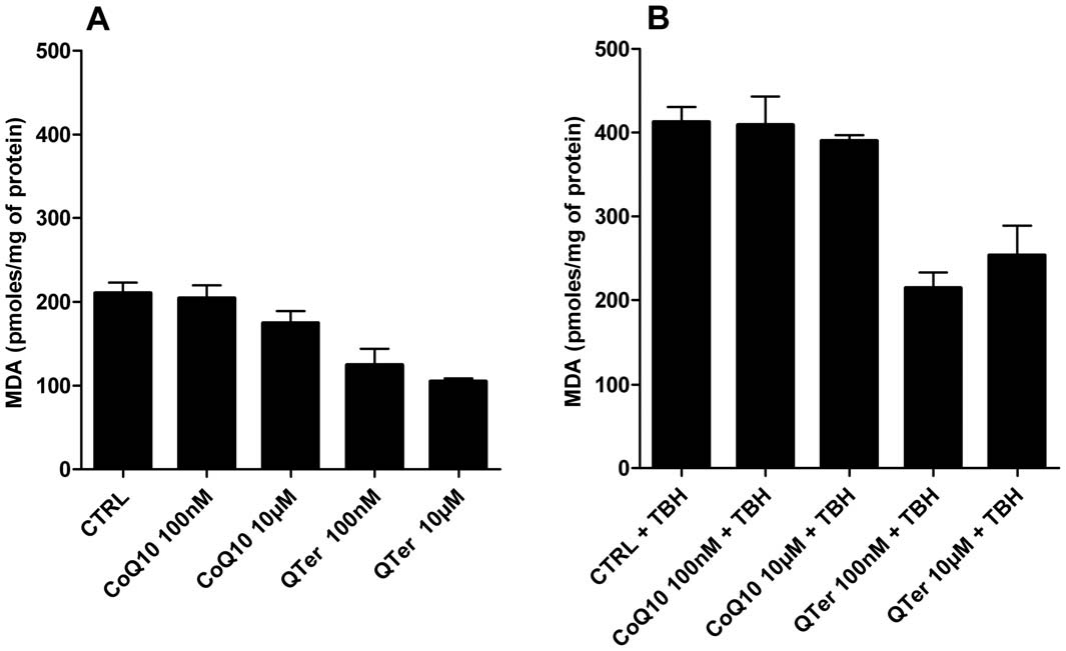
Malondialdehyde (MDA) levels in T67 cells treated with native CoQ_10_ or Qter. Cells were pre-treated for 24 hours with native CoQ_10_ and QTer (100 nM and 10 µM). Panel A shows the MDA levels in the absence of external oxidative stress. Panel B shows the MDA levels after 30 minutes exposure to 100 µM TBH. Data are the mean of two different experimental determinations and are normalized on total protein content.

Figure 5A and 5B report the MitoSOX Red staining of H9c2 cells without (Fig. 5A) and with (Fig. 5B) 100 nM Qter® pre-treatment for 24 hours: the lower staining observed in Figure 5B suggests that cellular CoQ_10_ supplementation reduces the ROS level also in absence of an oxidative insult. In Figure 5C is reported the MitoSOX Red fluorescence intensity obtained by Image J software analysis. ROS damage can be evaluated by measuring the presence of oxidative products such as malondialdehyde (MDA) and conjugated dienes. We observed that Qter treatment caused a statistically significant reduction of all lipid oxidation markers. Figure 6A and 6B show the MDA levels in T67 cells, both in absence (Fig. 6A) and in presence (Fig. 6B) of an oxidative insult, induced by treatment with 100 µM TBH. Even in this case it is possible to appreciate the higher efficiency of Qter® supplementation with respect to native CoQ_10_.

**Figure 7:**
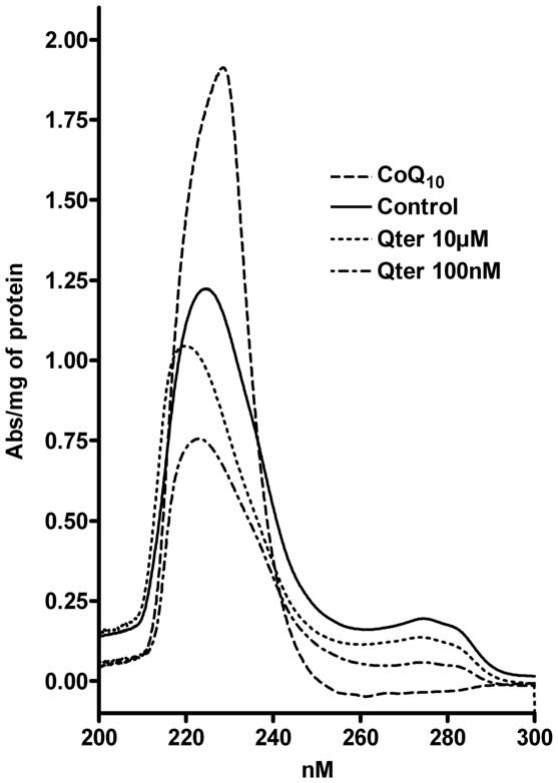
UV Spectra of conjugated dienes. Membrane lipids were extracted from T67 cells treated for 24 hours with Qter (100 nM and 10 µM) and CoQ_10_ (10 µM) after 30 minutes exposure to 100 µM TBH. Each spectrum was obtained as a difference spectra between TBH treated and TBH untreated samples. Spectra are normalized on total protein content and are representative of three different sets of experiments.

Figure 7 reports the differential absorption spectra of conjugated dienes extracted from T67 cells pre-treated with CoQ_10_ after 100 µM TBH exposure. The spectrum of cells treated with 100 nM Qter showed the lowest absorbance and the peak was shifted towards shorter wavelengths according to the presence of a lower conjugation status. On the other hand, the spectrum obtained by cells treated with 10 µM of native CoQ_10_ showed a higher absorbance and the peak was red shifted, indicating a high amount of conjugated dienes.

**Figure 8:**
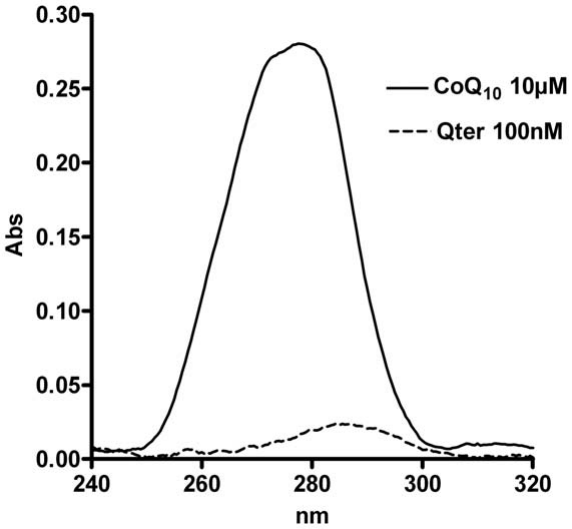
UV spectra of oxidized and reduced ubiquinone. T67 cells were treated for 24 hours with 100 nM Qter or 10 µM native CoQ_10_, then ubiquinone was immediately extracted, from an equal number of cells, with isopropyl alcohol (for further details see Materials and methods) and the UV spectra were recorded between 320 and 240 nm. The ubiquinone extracted from 100 nM Qter treated sample appears to be completely reduced with a maximum absorption peak at 290 nm, while the ubiquinone extracted from 10 µM native CoQ_10_ treated sample has a maximum absorption peak shifted towards 275 nm, indicating the presence of the ubiquinone oxidized form. Spectra are representative of three different experiments.

The oxidative stress observed in cells treated with high amounts of native CoQ_10_ can be due to an incomplete reduction of the supplemented quinone. Figure 8 shows the absorption spectra of CoQ_10_ extracted from T67 cells treated with 10 µM native CoQ_10_ or 100 nM Qter. The spectrum obtained from 100 nM Qter treated cells showed a maximum close to 290 nm, indicating that ubiquinone is mainly present in the reduced form. When cells were treated with 10 µM of native CoQ_10_, the spectrum was broad and the maximum was shifted towards 275 nm, indicating the contemporary presence of oxidized and reduced quinone forms.

### Mitochondrial membrane potential

**Figure 9:**
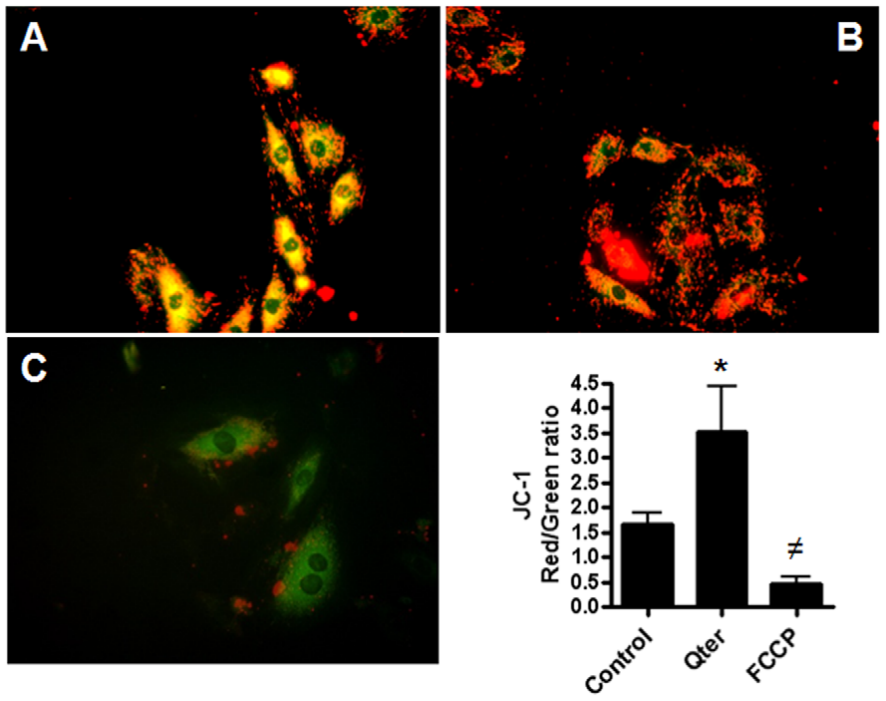
Assessment of mitochondrial potential by JC-1 staining. Representative images show JC-1 fluorescence in control H9c2 cells (A), H9c2 cells treated for 24 hours with 100 nM Qter® (B) and H9c2 cells treated with 500 nM of the uncoupler FCCP (C). Panel D shows quantitative analysis of Red/Green fluorescence ratio measured by ImageJ software. (n = 20, * p≤0.001 vs. control; ≠p≤0.001 vs. control.).

To assess whether Qter® administration could alter mitochondrial membrane potential, JC-1 fluorescence assay was performed in H9c2 cells. In control cells with normal mitochondrial membrane potential, JC-1 accumulates in mitochondria as aggregates with a red fluorescence emission while the monomeric form is prevalent in the cytoplasm with a green fluorescence emission (Figure 9A).

In Qter treated cells, JC-1 probe was mainly in the aggregated state resulting in a higher red/green fluorescence ratio, suggesting a higher incorporation of the probe into mitochondria as a consequence of a higher membrane potential(Figure 9B). Treatment with 500 nM FCCP prevent JC-1 mitochondrial incorporation, resulting in a pronounced green fluorescence due to the complete loss of mitochondrial membrane potential (Figure 9C). The red/green fluorescence ratios are summarized in Figure 9D.

## Discussion

Coenzyme Q_10_ is a lipid-soluble compound mainly found in mitochondria. It is mostly endogenously produced within cells though small amounts can be provided by food intake. Analysis of CoQ_10_ subcellular distribution shows that a large portion of CoQ_10_ (40–50%) is localized in the mitochondrial inner membrane, with smaller amounts in the other organelles and in the cytosol. The high concentration of CoQ_10_ in the mitochondria reflects its important role in electron transport chain: age-related decrease in mitochondrial CoQ_10_ content is responsible for oxygen consumption decline).

From a physiological point of view, tissue CoQ_10_ content is subject to regulation by several factors including oxidative stress and aging [1], [22].

Supplementation with CoQ_10_ has been thought to be beneficial, especially for situations in which adequate CoQ_10_ production is adversely affected [6]. A large number of clinical studies have evaluated the effects of CoQ_10_ supplementation on oxidative stress, both in physiological or pathological conditions.

The results obtained *in vivo* about CoQ_10_ tissue distribution are quite controversial. Ibrahim et al. (Ibrahim et al., 2000) [23] observed that CoQ_10_ oral administration did not alter the levels of this compound in the heart. Furthermore there is no evidence so far showing that dietary CoQ_10_, which is found to increase the CoQ_10_ content in lipoproteins and in the liver, is taken up by other tissues under normal conditions. These uncertain results could be partially attributed to the poor water solubility of CoQ_10_ that impairs its intestinal absorption, tissue distribution and mitochondrial incorporation.

The aim of the present study is to evaluate the *in vitro* efficacy of CoQ_10_ supplementation in improving mitochondrial function and protection against oxidative stress. Cells supplemented with CoQ_10_ do not often show any improvement in their bioenergetics status.

These negligible effects can be explained by several factors, first of all its strong lipophilic nature that results in its accumulation in extra-mitochondrial membranes [24], [25] while only a small portion (∼11%) can reach the mitochondria [26], [27]. The exogenous CoQ_10_ found in mitochondria is likely to be localized in the outer membrane, thus it is not available to the respiratory chain [28], [29].

Our data showed that Qter® has a better cellular uptake and mitochondrial incorporation compared to native CoQ_10_: from 10 to 100 fold lower concentrations are required to achieve similar cellular and mitochondrial CoQ_10_ amount.

A better uptake is the first step to proper CoQ_10_ insertion into biological membranes and in particular for a significant incorporation in the inner mitochondrial membrane (IMM). We can assume that Qter® promotes a correct CoQ_10_ insertion into the IMM since an increase in mitochondrial respiration, ATP production, mitochondrial membrane potential and protein synthesis are observed in the cell lines tested. In an interesting paper by Somayajulu et al. similar results were reported using a different water soluble CoQ_10_ formulation in human neuroblastoma cells (SH-SY5Y) and teratocarcinoma cells (NT2); in particular the authors described a protective effect of CoQ_10_ treatment on mitochondrial potential, ATP levels and oxidative stress after hydrogen peroxide exposure [30].

The great importance of a correct insertion is well explained by our data: cells treated with 10 µM of native CoQ_10_ present a mitochondrial ubiquinone concentration close to the one observed in cells treated with 100 nM Qter®, but the bioenergetic effects are quite different.

Moreover lipid peroxidation induced by an oxidative insult is reduced in 100 nM Qter® treated cells as shown in Figure 6B and Figure 7.

For this reason the chemical formulation of CoQ_10_ may play a crucial role in determining the correct integration of the molecule in the mitochondrial membrane. Nevertheless an increased content of CoQ_10_ in the mitochondrial membrane does not necessarily imply an automatic increase in mitochondrial function (Table 3). It is well known that respiration rate and ATP synthesis are highly regulated processes that are affected by many factors, primarily cell energy requirements.

Respiration data reported in Table 2 suggest that the mitochondrial ubiquinone content can affect the oxygen consumption under high energy requirement conditions (e.g. high ADP content). In this condition the oxygen consumption rate increases and the CoQ_10_ content could became the rate limiting factor. A similar behavior is observed in intact cells under FCCP uncoupled condition; in fact, the reported values of endogenous respiration rate were most likely due to an intermediate respiration state (state4/state 3 mixed state) in which the rate-limiting step was not affected by CoQ_10_ addition (Table 1).

Our data show that a high respiration rate is positively correlated with the increased amount of mitochondrial CoQ_10_, suggesting that its supplementation can play an important role in diseases related to CoQ_10_ deficiency (aging, Parkinson Disease, Alzheimer and mitochondrial myophaties). These data correlate with increased NADH-Cyt.c and Succinate-Cyt.c reductase activity observed in Hl-60 cells treated with CoQ_10_ reported by Navas and co-workers [31].

The mitochondrial respiratory chain organization could play an important role in the increase of respiratory activity. Currently, two models have been proposed: the random collision model [32] and a supercomplex organization called Respirasome [33]. In the first model the electron transfer through the respiratory chain is assured by free diffusion of each component within the IMM. In this scenario, CoQ_10_ forms a pool used by all the CoQ-dependent respiratory Complexes (mainly Complex I, II and III). On the other hand, the Respirasome requires a solid state organization in which only bound CoQ_10_ is involved in electron transfer. This last hypothesis seems to be in contrast with a dose dependent effect of CoQ_10_ addition on the respiratory rate. However, it may be possible that the bound ubiquinone should be in equilibrium with the pool. This hypothesis could explain the beneficial effect of exogenous CoQ_10_ supplementation [34].

Nevertheless, treatment with high doses of ubiquinone, despite of its formulation, induces a loss of sensitivity to uncoupling agents and increases oxidative stress. We can argue that an excessive incorporation of CoQ_10_ may perturb the lipid environment of cellular membranes while oxidative stress may be due to the excess of ubiquinone that remains in its oxidized form. For this reason it is not recommended to treat patients with high doses of coenzyme Q_10_. In particular, our results show that it is necessary to use from 10 to 100 fold concentrations of native CoQ_10_ to achieve comparable amounts of ubiquinone respectively in whole cells or isolated mitochondria.

The antioxidant role of the CoQ_10_ reduced form is well known. It localizes in cellular membranes where it acts as a ROS scavenger together with vitamin E. Cells treated with 100 nM Qter® appear to be more resistant to oxidative stress; in fact, Figure 8 shows that in this condition the quinone is mainly in the reduced form

The higher Qter® efficiency is mainly due to its greater water solubility. In fact, compared with native CoQ_10_, Qter® is about 200 times more soluble in water, while retaining its antioxidant capacity [35], [36].

### Conclusion

Our *in vitro* study underlines important issues regarding CoQ_10_ treatment. Present results demonstrate that adequate channeling of CoQ_10_ is important to ensure proper cellular uptake. The vehicle used to terclatrate CoQ_10_ maintains it in a monomeric form, that results in a correct insertion into membranes, in particular in the inner mitochondrial membrane. The improved bioavailability allows treatments with low doses of ubiquinone that prevent unspecific accumulation with deleterious effects on cell viability.

Although CoQ_10_ supplementation has shown beneficial effect in many physiopathological alterations, there are few experimental evidences of a direct improvement of mitochondrial functions after CoQ_10_ treatment. Some interesting papers by Somayajulu and McCarthy describe the protective effect of a water soluble CoQ_10_ in *in vitro* and *in vivo* studies [30], [37], [38].

We demonstrated that increased mitochondrial ubiquinone content results in a general improvement of bioenergetic parameters, like oxygen consumption, ATP content, mitochondrial potential and protein synthesis.

Recently, the beneficial effect of terclatrated CoQ_10_ supplementation in vivo, both in animal models [39], [40] and humans [41] has been reported. Thus, this work represents a strong rationale for the clinical use of Coenzyme Q_10_ and highlights the enhanced biological effects of Qter® that make it the eligible CoQ_10_ formulation for ubiquinone supplementation in patients.

## Materials and Methods

### Reagents

All chemicals used throughout the present study were of the highest analytical grade, purchased from Sigma-Aldrich, unless otherwise specified. Dulbecco's modified Eagle's medium, trypsin, penicillin, streptomycin and fetal bovine serum were purchased from Invitrogen. Qter® was supplied by Scharper Therapeutics S.r.l. (Milan, Italy). Native CoQ_10_ was from Kaneka, Japan.

### Drug preparation

Qter® is described in the patent number WO/2003/097012 by Actimex S.r.l. CoQ_10_ is 10% (w/w) of Qter® formulation. Qter® concentration refers to the CoQ_10_ amount into the multicomposite material. Qter solution was freshly prepared dissolving Qter in DMEM at 100 nM or 10 µM CoQ_10_ final concentration.

Native CoQ_10_ stock solution was prepared in ethanol at 5 mM concentration and diluted with DMEM to final concentration of 100 nM or 10 µM prior to use.

### Cell culture

The T67 human glioma cell line was derived by Lauro et al. [42] from a World Health Organization (WHO) Grade III gemistocytic astrocytoma. H9c2 embryonal rat heart-derived cells were obtained from European Collection of Cell Cultures, ECACC. Cells were cultured in Dulbecco's modified Eagle's medium (DMEM), supplemented with 10% fetal bovine serum (FBS), 100 UI/ml penicillin, 100 µg/ml streptomycin, and 40 µg/ml gentamycin, in a 5% CO_2_ atmosphere at 37°C, with saturating humidity. Cell viability and number were measured by trypan blue exclusion method [43].

### Preparation of Mitochondria Fractions

Mitochondria were isolated according to procedures previously described [44].

### Extraction and quantification of Coenzyme Q

Treated cells were carefully washed with PBS before extraction procedures. Extraction of coenzyme Q from cells and isolated mitochondria was performed as described by Takada et al. [45]. Quantification of CoQ_10_ was performed by HPLC analysis. 50–100 µl of ethanolic extract was chromatographed on a C18 column (Kinetex, Phenomenex, 2.6 µm, 100×4.6 mm), using a mobile phase consisting of ethanol: water (97∶3, v/v) at a flow rate of 0.6 ml/min. The concentrations of CoQ_10_ were obtained by comparison of the peak areas with those of standard solutions. Data are reported as the mean ± standard deviation of at least three independent experiments.

To evaluate the reduction state of ubiquinone, cells were treated for 24 hours with 10 µM of native CoQ_10_ or 100 nM Qter. After this time, T67 adherent cells were washed twice with PBS, then detached by trypsin-EDTA and centrifuged at 300×*g* for 3 min; then the pellet was resuspended in PBS, and centrifuged again. The pellet was resuspended in cold isopropyl alcohol and vortexed for 30 seconds, then centrifuged at 15000 g at 4°C for 5 minutes. The organic phase was transferred in a quartz cuvette and the UV spectrum was collected between 240 and 320 nm with a Jasco V-550 spectrophotometer.

### Cell permeabilization

Cells were permeabilized with digitonin according to Chomyn A. [39] and immediately used for polarographic assay [44], [46]. Cell number and permeabilization was measured by trypan blue exclusion method.

### Oxygen consumption

#### Intact cells

T67 and H9c2 cell lines were treated for 24 h at 37°C in 5% CO2 with 100 nM CoQ_10_ or Qter® in DMEM plus FBS. Intact cells (1×10^6^ cells) were assayed for glucose supported oxygen consumption at 30°C in DMEM using a thermostatically controlled oxygraph (Instech Mod.203).

#### Permeabilized cells

Cells were treated as above and assayed for oxygen consumption in respiration buffer (250 mM sucrose, 20 mM HEPES, 10 mM MgCl2, 1 mM ADP, 2 mM KH2PO4, pH 7.4) after permeabilization. Mitochondrial respiration (state 3 respiration) from complex I was started by adding 5 mM glutamate/malate (G/M) and then stopped with 2.5 µM Rotenone. Subsequently 12.5 mM succinate/glycerol-3-phosphate (S/G3P) was added to restart the respiration. In all experiments maximal respiration rate (uncoupled respiration) was achieved by adding 500 nM FCCP and oxygen consumption was completely inhibited by adding 4 µM Antimycin A at the end of the experiments.

### Reactive oxygen species (ROS) detection

H9c2 and T67 cells were seeded in 24-well plates at 4×10^4^ cells/well. After 24 h incubation at 37°C in 5% CO2 in culture medium supplemented with 100 nM CoQ_10_ or Qter®, cells were washed with phosphate buffered saline (PBS) and treated for 48 h with 100 nM Rotenone. Alternatively cells were treated for 30 minutes with 100 µM tert-butyl hydroperoxide (TBH) in PBS. Subsequently, cells were washed with PBS and treated with 10 µM DCFDA (2′,7′-dichlorofluorescein diacetate, DCFH-DA) in DMEM for 30 minutes, then washed again with PBS and the fluorescence increase in each well was measured (λexc = 485 nm; λem = 535 nm) with a plate reader (Wallac Victor, Perkin-Elmer, USA). Data are reported as the mean ± standard deviation of at least three independent experiments. In a separate set of experiments, basal oxidative stress in H9c2 cells was measured using the mitochondrial superoxide indicator MitoSOX Red. Cells were treated with 100 nM Qter® for 24 hours at 37°C in 5% CO2, then 5 µM MitoSOX was added. After 20 minutes of incubation, cells were washed twice with PBS and images were obtained using an IX50 inverted fluorescence microscope (Olympus, Tokyo) at 20× magnification. Fluorescence intensity was quantified by Image J software (NIH).

### ATP content

Intracellular ATP level was measured using luminescence ATP detection assay (ATPlite, PerkinElmer, USA) according to manufacturer's instructions. Data were reported as arbitrary luminometric units, measured with the microplate reader Wallac Victor multilabel counter and normalized to total protein content, determined by Lowry method [47]. Alternatively, intracellular ATP was measured by HPLC method. ATP was extracted essentially as described by Strehler et al. [48]. Distilled water (180 µl) preheated to 100°C was added to cellular samples in Eppendorf tubes and boiled for 5 min with occasional vortexing. Tubes were transferred to ice until HPLC analysis. A mobile phase containing 100 mM K2HPO4 (pH 5.75), 0.1% TBAF (tetrabutylammonium fluoride) and 2% acetonitrile was pumped through a Kinetex C18 (Phenomenex) column at ambient temperature at a flow rate of 0,6 ml/min [49]. Absorbance at 254 nm was monitored by a photodiode array detector (Waters 996). ATP peak was identified by its retention time and by using co-chromatography with standard.

### Thiobarbituric acid assay of malondialdehyde and lipid-conjugated dienes assay

T67 cells were treated for 24 h at 37°C in 5% CO_2_ with native CoQ_10_ (10 µM and 100 nM) or Qter® (10 µM or 100 nM) in DMEM plus FBS and washed with PBS before treating for 30 minutes with 100 µM tert-butyl hydroperoxide (TBH) in PBS.

After TBH treatment, adherent cells were washed twice with PBS, then detached by trypsin-EDTA and centrifuged at 300×*g* for 3 min; then the pellet was resuspended in PBS and centrifuged again.

Quantification of thiobarbituric acid reactive substances (TBARS) was carried out as described by Buege and Aust [50].

The formation of conjugated dienes, in T67 cells treated as above was assayed according to Buege and Aust [50].

### JC-1 stain for mitochondrial membrane potential (Δψm) measurement

The fluorescent probe JC-1 (5, 5′, 6, 6′-tetrachloro-1, 1′, 3, 3′-tetraethylbenzimidazol carbocyanine iodide) was used to measure the mitochondrial membrane potential (Δ*ψ*) of H9c2 cells. JC-1 is a cationic dye that is accumulated in mitochondria following membrane potential.

At low concentrations the probe is present in monomeric form, with green fluorescence emission (525±10 nm), but at higher concentrations it forms J-aggregates after accumulation in the mitochondrion, with red fluorescence emission (590±10 nm).

After incubation with 10 µM JC-1 at 37°C for 10 min, the culture medium containing JC-1 was removed. Cells were washed twice with PBS, and analyzed by IX50 fluorescence microscope (Olympus, Tokyo).) at 20× magnification. Fluorescence intensity was quantified by Image J software (NIH). Mitochondrial depolarization was achieved by treating cells with 500 nM FCCP, indicated by a decrease in the red/green fluorescence intensity ratio

### Statistical analysis

Statistical analysis of data was performed with Student's t-test using GraphPad Prism software.
